# Emerging Materials for Interfacial Solar‐Driven Water Purification

**DOI:** 10.1002/anie.202214391

**Published:** 2023-01-02

**Authors:** Sijia Cao, Arne Thomas, Changxia Li

**Affiliations:** ^1^ Department of Electrochemical Energy Storage Helmholtz-Zentrum Berlin für Materialien und Energie Hahn-Meitner-Platz 1 14109 Berlin Germany; ^2^ Institute of Chemistry University of Potsdam 14476 Potsdam Germany; ^3^ Department of Chemistry, Functional Materials Technische Universität Berlin Hardenbergstraße 40 10623 Berlin Germany; ^4^ Department of Inorganic Chemistry - Functional Materials Faculty of Chemistry University of Vienna Währinger Straße 42 1090 Vienna Austria

**Keywords:** Covalent Organic Frameworks, Desalination, Metal-Organic Frameworks, Polymers, Solar-Driven Water Purification

## Abstract

Solar‐driven water purification is considered as an effective and sustainable technology for water treatment using green solar energy. One major goal for practical applications is to improve the solar evaporation performance by the design of novel photothermal materials, with optimized heat localization and water transport pathways to achieve reduced energy consumption for water vaporization. Recently, some emerging materials like polymers, metal‐organic frameworks (MOFs), covalent organic frameworks (COFs) and also single molecules were employed to construct novel solar evaporation systems. In this minireview, we present an overview of the recent efforts on materials development for water purification systems. The state‐of‐the‐art applications of these emerging materials for solar‐driven water treatment, including desalination, wastewater purification, sterilization and energy production, are also summarized.

## Graphical abstract

Research on solar‐driven water purification has grown rapidly over the past years and emerging materials such as polymers, MOFs and COFs have been employed in this field recently. This minireview gives an overview of the basic concepts and principles for solar evaporation systems and on the development of novel photothermal materials.

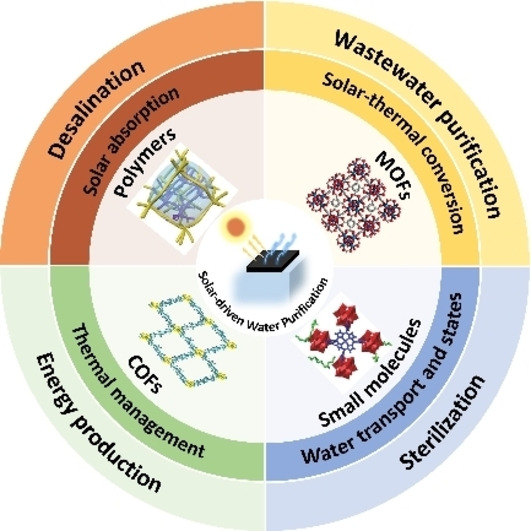

## 1 Introduction

Drinkwater shortage has become a global concern due to the inherent freshwater scarcity on earth and water pollution caused by industrial/agricultural production and domestic sewage.^[1]^ Notably, under the current Covid‐19 pandemic, medical contaminants, especially the disinfection byproducts, which are potentially carcinogenic and cause a high risk of microbial resistance, are a further threat to water security. Drinkable water shortage have been reported to affect two‐thirds of the global population.^[1]^ Some evaporation techniques, such as multistage flash and multiple effect distillation, are widely used to produce fresh water in the industrial desalination. However, these techniques require intensive heat and electricity, for example, 13–26 kWh for 1 m^3^ freshwater production.^[2]^ Therefore, developing effective methods for freshwater production with low energy consumption for water treatment is an urgent issue. Inspired by water circulation in nature, solar‐driven water treatment offers an opportunity to recover clean water from sea‐ or wastewater. Using green and sustainable solar energy as the only input power, this technology lowers carbon emission considerably and shows great prospects, especially for remote regions lacking electricity and infrastructures.^[3]^ Solar membrane distillation, a technique based on the traditional membrane distillation assisted by solar energy, have been well developed on large scale with considerable water production yield. However, the necessary setups can be quite complex and the solar conversion efficiency is usually low.^[4]^ Solar‐driven water purification has recently attracted great attentions, which comprises two processes: solar vapor generation (SVG) and water vapor collection. As one fundamental process, SVG involves the vaporization of liquid water at a much lower temperature than the boiling point. However, the natural vaporization of bulk water is a very slow process and the rate is usually lower than 0.3 kg m^−2^ h^−1^ under 1 kW m^−2^ (one sun irradiation). Photothermal materials have thus been utilized to enhance the evaporation rates. In general, water is soaked into the photothermal material and then evaporated by the heat produced from the material under sunlight irradiation. Systems for solar evaporation were however first developed in form of volumetric heating, using e.g. homogeneously dispersed metal nanoparticles (NPs) as solar absorbers, which convert the incident photons to thermal energy to heat the aqueous dispersion medium. These systems however require a long‐term and intense light source to heat the bulk water, resulting in a low light‐utilizing efficiency.^[5]^ To address these issues, solar‐driven interfacial evaporation was proposed to localize the heat at the water‐air interface, which can improve the efficiency even under lower irradiation intensity.^[6]^ Using this approach, the solar evaporators are usually located at a water reservoir to selectively heat the water reaching the evaporator surface under irradiation, which remarkably decreases the water volume which has to be heated. Additionally, in such interfacial systems, the evaporation performance can be effectively tuned by means of the evaporator structure. Solar‐driven interfacial evaporation shows great potential to create practical and portable systems, for facile, cheap and decentralized water purification.

Comprehensive reviews have elaborated on the topics of photothermal materials,^[7]^ the rational design and architecture of solar evaporation systems,^[8]^ as well as strategies to enhance light absorption and SVG rates.^[9]^ Recently, some novel materials have attracted interest for solar‐powered water purification, however, these materials are rarely discussed in the mentioned reviews. In this context, we particularly focus here on the progress of newly developed materials, such as polymers, metal‐organic frameworks (MOFs), covalent organic frameworks (COFs), and some unique small molecules for interfacial solar‐driven water purification.

## 2 Principal Properties of SVG Systems

In an interfacial SVG system, the following four key principles should be optimized.

### 2.1 Solar Absorption

As essential component of SVGs, diverse solar absorbers have been applied, such as plasmonic metallic NPs (e.g., Au),^[5]^ inorganic semiconductors (e.g., Ti_2_O_3_),^[10]^ carbons (e.g., carbon nanotubes (CNTs), graphene),[^7f^, ^11^] conjugated polymers (e.g., polypyrrole (PPy)),^[12]^ and hybrid materials therefrom. Superior solar absorbers are desired to enable efficient absorption of solar irradiation covering a broad solar spectrum in the range from 250 nm to 2500 nm (AM 1.5 reference spectrum), simultaneously with minimal transmittance and reflectance.^[7b]^ The capability of light harvesting can also be enhanced by regulating the surface morphology to reduce light reflectance.[^12c^, ^13^]

### 2.2 Solar‐Thermal Conversion Ability

The mechanisms for light‐to‐heat conversion are related to the nature of the materials. The photothermal conversion ability can be controlled via varying band gaps, introducing defects sites, changing morphologies or creating composites.[^7b^, ^14^] The solar‐thermal conversion efficiency *η*_s‐t_ is a standard metric to evaluate the performance of solar‐radiation‐to‐thermal‐energy conversion. It can be estimated by the equation *η*_s‐t_ *=m* *h*_LV_/*C*_opt_ *P_0_* at the steady state, where *m* denotes the net evaporation rate, *h*_LV_ is the equivalent evaporation enthalpy of water through the solar evaporators, *P*_0_ represents the intensity of the incident radiation and *C*_opt_ is the optical concentration (i.e., *C*_opt_ *P*_0_ refers to the power density *Q_s_* of the incoming light illumination).^[12d]^

### 2.3 Thermal Management

Ideally, the converted thermal energy should be utilized entirely for heating the water. However, in a typical system, heat loss is inevitable, which takes place through three routes: downward conduction loss to bulk water, upward convection to the environment and radiation loss. Minimizing the thermal loss is crucial to ensure heat localization at the evaporation interface and promoting evaporation efficiency. Due to the high thermal conductivity of the water itself (0.598 W m^−1^ K^−1^, 20 °C), the downward conduction dissipation is a main loss factor and related to the thermal conductivity of the materials.^[7b]^ To settle the problem, thermal‐insulator‐supported bilayer structures are applied to spatially separate the solar evaporators from the bulk water.^[15]^ Besides, application of nanostructures^[12a]^ and the spatial distribution of solar absorbers^[16]^ have been demonstrated to affect the heat confinement.

### 2.4 Water Transport and States

Water transport depends on the porosity and polarity of the evaporators. Highly porous structures can accommodate large amounts of water molecules and provide sufficient pathways for water transport.[^12a^, ^17^] Hydrophilic parts in the solar evaporators can interact with water molecules, hence facilitating the rapid transport from bulk water to the evaporator surface.^[18]^ However, small pores and very hydrophilic channels might bind adsorbed water too strongly to enable rapid evaporation, thus pore size, surface area and polarity are all factors which need to be controlled to enable efficient SVG systems.^[17]^ Water molecules can interact with the evaporator networks via strong hydrogen bonding and electrostatic interactions to form bound water (BW), which is thus not accessible for water evaporation. In contrast, the residual water molecules within the pores behave like bulk water, also called free water (FW). There is also an intermediate region between BW and FW called intermediate water (IW).^[19]^ In general, the ratio of IW to FW determines the rate of water evaporation. This ratio can be identified by Raman spectroscopy.[^12c^, ^18^]

Overall, in order to evaluate the performance of SVG systems, light absorptivity, solar‐thermal conversion efficiency *η*_s‐t_, evaporation rates, the thermal conductivity and equivalent vaporization enthalpy have been identified as the main performance indicators.

## 3 Emerging Materials

### 3.1 Polymer‐Based Evaporators

Polymeric materials are ideal candidates for the construction of self‐floating solar evaporators because of their low density and processibility. Depending on the synthesis route and manufacturing, they can possess mechanical stability, porous structures, low thermal conductivity and adjustable chemical functionalities.^[20]^

**Figure 1:**
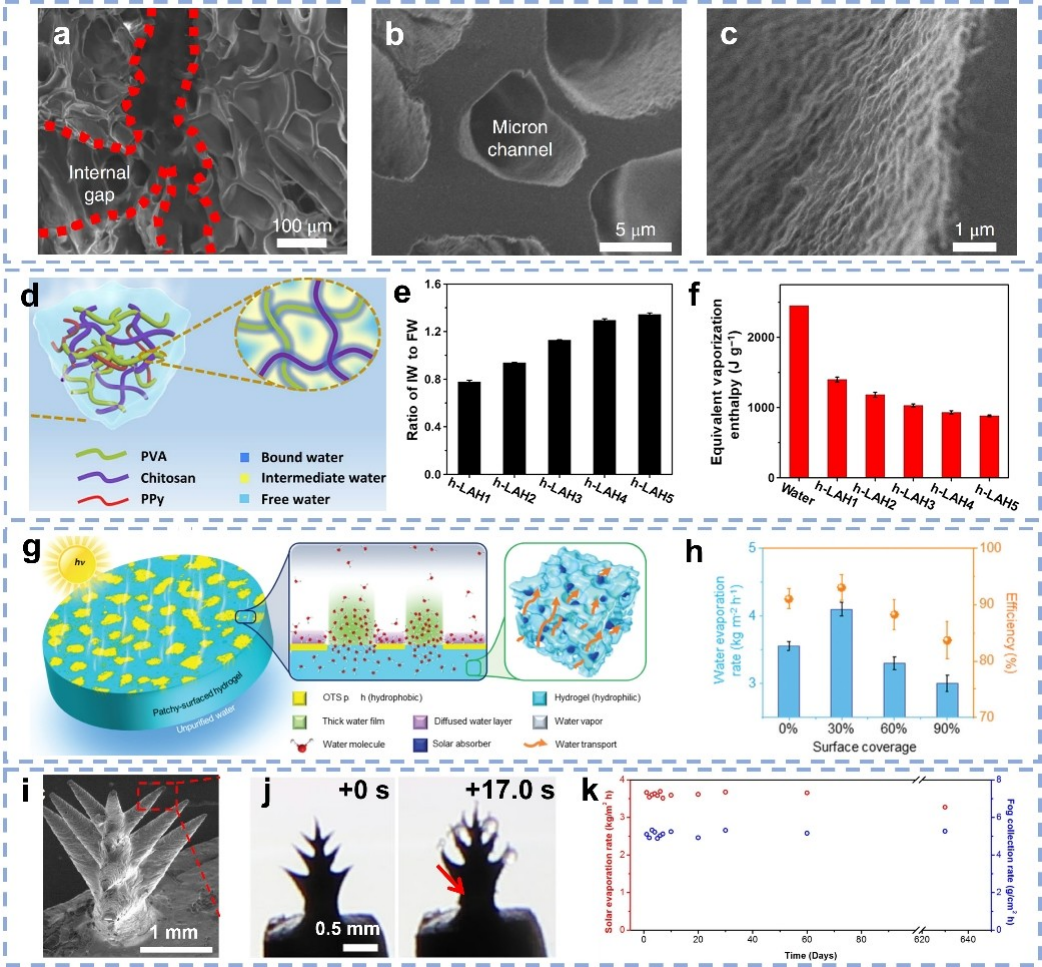
SEM images of PVA/PPy hydrogels showing a) internal gaps, b) micron channels and c) the wrinkled internal surface.^[12a]^ d) Cross‐linked PVA/PPy/chitosan polymer network and the water states inside the hydrogels. e) The ratio of IW/FW. f) The equivalent water vaporization enthalpy of bulk water and water inside the hydrogels.^[18c]^ g) Illustration of surface‐modified hydrogels. h) SVG rates and corresponding energy efficiency varying surface coverage under one sun.^[13b]^ i) SEM image of one branch micro‐tree in the PVA/PPy gel membrane. j) Snapshots of fog collection process for a single gel tree. k) Stable dual water harvesting functions over 20 months.^[12c]^

As low‐cost, biocompatible and easy‐processable polymer, poly (vinyl alcohol) (PVA) showing high affinity to water molecules, has become the most popular polymer to fabricate solar evaporators, usually in the form of hydrogels. As PVA does not absorb light in the visible, various absorbers, for instance, PPy,[^12a^, ^12b^] activated carbon (AC),^[13a]^ or Ti_2_O_3_ NPs^[10]^ were embedded into the PVA networks during the gelation process to form the solar hydrogel evaporators. In 2018, Yu's group first developed PVA‐based hydrogels as solar evaporators with interpenetrating PPy chains by in situ polymerization of pyrrole.^[12a]^ Compared to the pure PVA networks, the PVA/PPy interconnected hydrogels possessed highly hierarchical structures, minimizing thermal loss and optimizing water replenishment in the capillary channels (Figure 1a–c). Such hydrogels thus displayed a high evaporation rate of 3.2 kg m^−2^ h^−1^ under one sun, ten times higher than for pure water. Furthermore, chitosan was introduced into PVA/PPy hydrogels (Figure 1d) to regulate the ratio of IW to FW and thus the required energy for water evaporation (Figure 1e and f), yielding SVG rates of up to ≈3.6 kg m^−2^ h^−1^.^[18c]^ Additionally, polystyrene sulfonate (PSS) was used to modify the evaporators and control the affinity to water molecules.^[18c]^ By uniformly interpenetrating the PSS chains into PVA hydrogels, the IW content could be increased to 50 %, which enabled a superior SVG rate of ≈3.9 kg m^−2^ h^−1^. Evaporator surfaces have been further engineered to improve the SVG performance.[^12c^, ^13^] PVA hydrogels with AC as solar absorber and a dimpled surface showed better capability to localize the light as comparable ones with flat surface.^[13a]^ Furthermore, chemical modification of the evaporator surface using hydrophobic trichloro(octadecyl)silane to control the amount of hydrophobic/hydrophilic regions was proposed.^[13b]^ Water is confined at the hydrophilic regions and thus the evaporation takes place in these regions instead of over the whole surface (Figure 1g). By changing the surface coverage of hydrophobic compounds, hydrogels with a rate of ≈4.0 kg m^−2^ h^−1^ with 93 % efficiency under one sun (Figure 1h) have been produced. Recently, Shi et al. designed a PVA/PPy gel membrane with microtree‐shaped surface using 3D printing.^[12c]^ Each tree on the membrane surface was ≈4 mm tall with a ≈0.8 mm bottom diameter and ≈20 μm branch tips (Figure 1i). Such structures enabled effective light harvesting and enlarged the evaporation surface area, displaying a SVG rate of 3.64 kg m^−2^ h^−1^ under one sun. Remarkably, the membranes could capture fog at nighttime with a rate of 5.0 g cm^−2^ h^−1^, proving their potential as an all‐day water harvester (Figure 1j and k).

Very recently, tuning of channel size to control water transport rates was emphasized to influence the SVG performance in PVA hydrogels.^[21]^ Freeze‐drying is usually used to create micron‐sized channels in solar evaporators; however, these channels show high tortuosity which can hamper the water transport. Guo et al. utilized PMMA particles as template to create uniform and size‐controlled channels in PVA‐based evaporators.^[21a]^ The water transport rate increased with increasing channels size, suggesting its strong dependency on the pore size. Through the optimization of the channels, the evaporators showed more effective water transport and thus 40 % accelerated SVG rates compared to control samples prepared by freeze‐drying. Considering more realistic environmental conditions, measurements of SVG rates under weak irradiation have received attention recently.^[22]^ By imitating the structure of natural leaves and applying reduced carbon dots (rCD) as solar absorbers, Tu et al. developed SVG evaporators from PVA/rCD spherical microgels with diameters ranging from ≈130 nm to ≈27 μm.^[22a]^ The authors state that each microgel function as an artificial mesophyll cell, which could effectively evaporate the water film surrounding each microgel. Abundant gel units can additionally harvest thermal energy from the environment through tiny liquid–gas interfaces to enhance the evaporation rates. Indeed, a SVG rate of 2.18 kg m^−2^ h^−1^ is achieved under 0.5 sun, which even surpasses the most reported SVG yields under one sun. Extracting the energy from environments would be a rational and effective guidance in the construction of solar evaporators.

**Figure 2:**
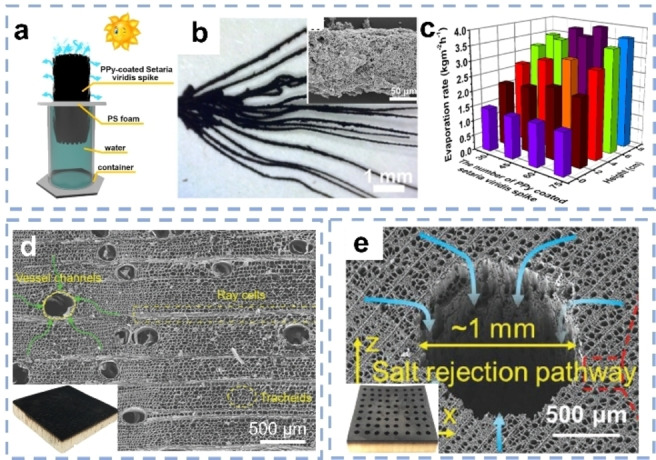
a) Scheme of the PPy coated Setaria viridis as solar evaporator. b) Photograph and SEM image of the spikes coated with PPy.^[12e]^ c) SVG rates with different height and number of spikes. SEM images showing the multi‐dimensional channels of d) balsawood‐based evaporators^[23]^ and e) drilled basswood‐based evaporators.^[24]^

Also cellulose materials have been exploited to construct SVG systems. Cellulose is the most abundant biopolymer on earth and therefore also of interest in terms of sustainability.^[25]^ Cellulose‐based materials, such as bacterial nanocellulose (BNC),^[15c]^ plant‐derived cellulose,[^12e^, ^23^, ^24^] commercial cellulose paper^[26]^ or cellulose nanofiber membranes (CNF),^[27]^ have been applied as hydrophilic substrates. Xie et al. coated PPy on the spikes of a natural plant, Setaria viridis (Figure 2a,b), creating a large evaporation surface.^[12e]^ By optimizing the height and number of spikes, the evaporators showed a SVG rate of 3.72 kg m^−2^ h^−1^ under one sun (Figure 2c). Cellulose materials can also function as thermal insulation layers. Jiang et al. deposited graphene oxide (GO) flakes into BNC hydrogels to fabricate bilayer evaporators. BNC not only provides water transport channels but also suppresses the heat dissipation to the underlying water.^[15a]^ Inspired by the water transportation of trees, wood materials with vertically aligned channels and hydrophilic walls are ideal platforms for solar evaporation. Hu and co‐workers found that natural balsawood exhibits a bimodal porous structure with both vessel channels (180 to 390 μm) and narrow racheid channels (18 to 39 μm).^[23]^ Surface‐carbonized balsawood showed good anti‐clogging ability in brine treatment when used as solar evaporator (Figure 2d). Subsequently, the authors drilled millimeter‐sized channels into the natural basswood (Figure 2e).^[24]^ The newly prepared large channels had a higher water flux under the same pressure gradient and lower salt concentration due to the multi‐directional mass transfer compared to the pristine wood. As a result, the modified evaporators displayed faster salt exchange and thus could effectively hinder the salt accumulation.

**Figure 3:**
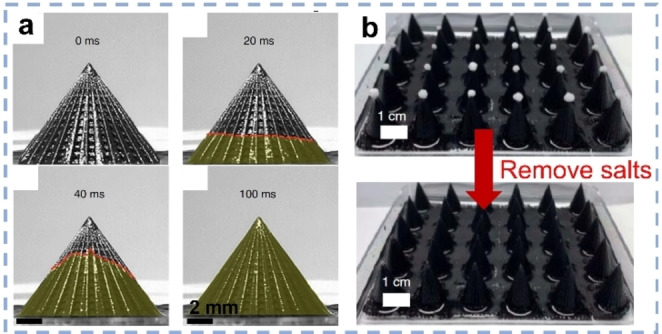
a) Time‐dependent water upward spreading process on a bird‐beak shaped 3D evaporator surface. b) Photographs showing the salt removal on these structures.^[11]^

Apart from widely applied PVA and cellulose materials, other polymers are also used to construct functional SVG systems. Polyvinylidene difluoride (PVDF),^[28]^ polyurethane (PU),^[29]^ poly(ether sulfone) (PES),^[30]^ polydimethylsiloxane (PDMS)^[31]^ or melamine foams,^[32]^ were used as substrates to build solar evaporators by incorporating versatile absorbers. Analogous to PPy, polyaniline (PANI)[^30^, ^33^] and PDA^[34]^ are often chosen as the photothermal material because of the broad solar absorption and good photothermal utilizing efficiency. Recently, a polyacrylate copolymer system with CNTs as solar absorber was utilized to manufacture solar evaporators with bird beak‐shape using 3D printing (Figure 3a).^[11]^ These materials displayed efficiency of ≈96 % and a SVG rate of 2.63 kg m^−2^ h^−1^ under one sun. By optimizing the height‐to‐diameter ratio, the intriguing structures could dramatically accelerate water evaporation even without illumination, which gained an astonishing evaporation rate of 1.17 kg m^−2^ h^−1^ under dark conditions, the highest rate reported so far. Notably, the salt crystals concentrated on the beak‐tips during brine treatment and could be thus easily removed in solar desalination (Figure 3b).

### 3.2 MOF/COF‐Based Evaporators

Owing to their high surface area and porosity and their tunable structure and functionality, MOFs and COFs have been broadly investigated for water treatments such as organic contaminants adsorption/degradation, ion capture or seawater desalination.^[35]^ The group of Yaghi applied MOF‐801 [Zr_6_O_4_(OH)_4_(fumarate)_6_] to capture water from the atmosphere.^[36]^ The MOF‐801 based device can harvest 2.8 liters of water per kilogram of MOF under natural sunlight each day, even under low relative humidity (≈20 %). In recent years, MOFs and COFs were also developed for fabricating efficient SVG systems.

**Figure 4:**
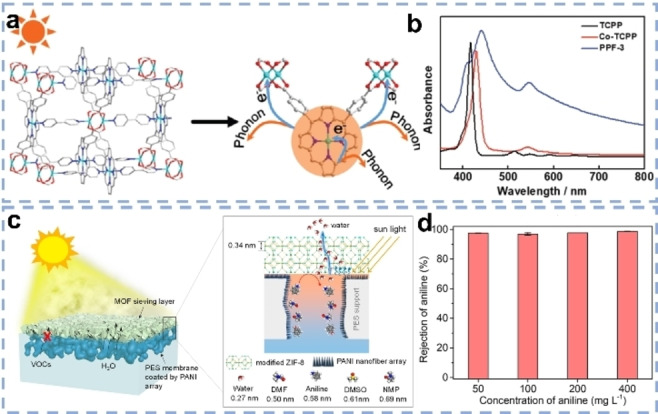
a) Scheme of the mechanism of the photothermal conversion of PPF‐3. b) Absorption spectra of pure TCPP and PPF‐3.^[28]^ c) Scheme of ZIF‐8‐based membrane for rejecting VOCs from water. d) Aniline rejections with different initial concentrations.^[30]^

MOFs, which are generated from metal ions or clusters and organic linkers, can easily crystallize under mild conditions with controllable morphology.^[37]^ Probably due to the sometimes low water stability, weak light absorption as well as the difficult processibility, MOFs are so far just rarely exploited for SVG. Nevertheless, recently, some MOFs were designed as photothermal materials.[^28^, ^38^] Chen et al. reported the synthesis of PPF‐3 from tetrakis (4‐carboxyphenyl) porphyrin (TCPP) and Co^2+^ as photothermal material.^[28]^ Such a MOF architecture enables efficient solar harvesting for two reasons: first, the highly porous and ordered structure of the MOFs allow for the deep penetration of photons to interact with a maximum amount of porphyrin molecules and second, metal sites as electron acceptors foster the non‐radiative relaxation of electrons for local heating (Figure 4a and b). Accordingly, when dispersed onto a PVDF membrane, PPF‐3 displayed a 1.99 kg m^−2^ h^−1^ SVG rate (70.3 % efficiency) under 2 sun, much higher than when just the porphyrin monomer was applied. Still, the sometimes‐observed low water stability impedes the applications of many MOFs to construct solar evaporators.[^30^, ^33^] Therefore, the coating of protecting layers was proposed. Li et al. applied PANI as protective layer and as solar absorber to encapsulate MOF [Cu_2_(OH)(BTC)(H_2_O)]_*n*_ (Cu‐BTC) nanorods.^[33]^ After growing Cu‐BTC nanorods on the PVDF substrates, the Cu‐BTC/PVDF membrane preserved its crystallinity for 3 days in water, while the PANI‐wrapped membrane was stable for two weeks even in seawater. Moreover, benefiting from the hydrophilicity, 1D nanochannels and a large evaporation surface area of Cu‐BTC, the hybrid membrane showed a SVG rate of 1.44 kg m^−2^ h^−1^ and 90.8 % efficiency under one sun.Most MOFs have pore sizes in the micropore range (pore diameter less than 2 nm), which is suitable for the adsorption and separation of small guest molecules.^[39]^ MOFs can function as molecular sieves to evaporate clean water from sewage containing volatile organic compounds (VOCs). Such VOCs and water can both evaporate und SVG conditionsand therefore solar‐driven evaporationis not an appropriate technique to purify polluted water containing VOCs. Peng et al. thus fabricated a composite membrane by growing a zeolitic imidazole framework‐8 (ZIF‐8) layer on a PANI‐coated PES membrane, where PANI worked as solar absorber layer (Figure 4c).^[30]^ ZIF‐8 has a larger aperture size (0.34 nm) than the kinetic diameter of water molecules (0.27 nm), but a smaller one than that of most VOCs (usually >0.5 nm), thus could reject VOCs from the evaporated steam. Furthermore, to improve the water stability, ZIF‐8 was modified by a partial ligand‐exchange reaction using a hydrophobic ligand, 5,6‐dimethylbenzimidazole. Ultimately, the hybrid membrane attained a high VOCs rejection efficiency up to 99 % at a SVG rate of 1.0 kg m^−2^ h^−1^ under one sun and could even treat a high‐concentration VOCs up to 400 mg L^−1^ (Figure 4d).

**Figure 5:**
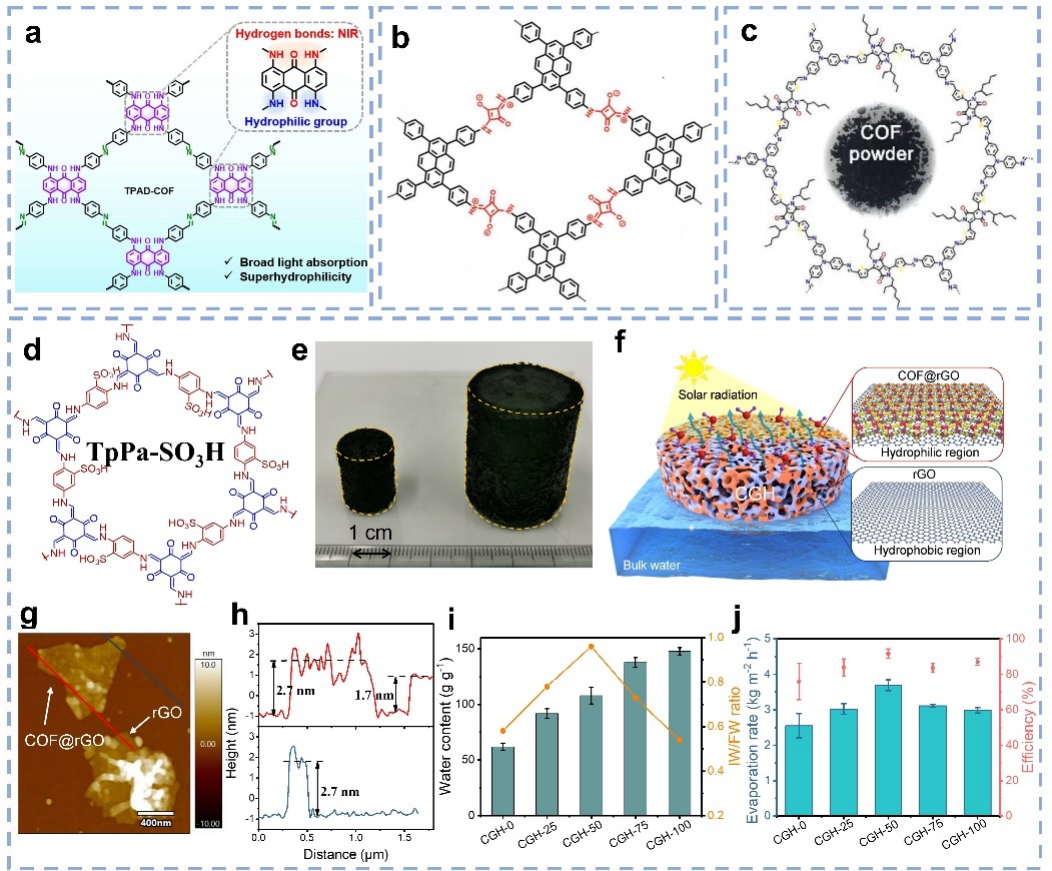
Chemical structure of a) TPAD‐COF,^[40]^ b) SQ‐linked COF,^[32]^ and c) DPP‐COF.^[41]^ d) Chemical structure of TpPa‐SO_3_H. e) Photograph of CGH‐50 synthesized using 20 mL (left) and 120 mL (right) autoclaves, respectively. f) Scheme of CGH for solar‐driven water evaporation. g) AFM image and h) the corresponding height profiles of CGH‐50. i) Saturated water content and the IW/FW ratio in CGHs. j) SVG rates and efficiency of CGHs.^[18a]^

COFs are emerging crystalline polymers formed via covalent bonds.^[42]^ In COF‐based SVG systems, COF powders are usually embedded in a porous matrix such as PVA gels,^[43]^ PDMS sponges,^[31]^ or polytetrafluoroethylene (PTFE) substrates.^[40]^ Furthermore, some COFs can be grown in situ on various substrates.[^18a^, ^44^] For example, Xia et al. reported porphyrin‐COFs grown on different substrates, e.g., wood, AAO membranes, fabrics via a one‐pot synthesis.^[44]^ As photothermal materials, the extended π‐conjugated system in COFs can facilitate π‐π* electronic transitions, resulting in broad light absorption. Relaxation to the ground state is then accompanied by thermal release.^[7c]^ However, the absorption of COFs usually just covers the lower wavelength parts of the visible solar spectrum. Applying monomers containing specific dye molecules is an effective approach to extend the light absorption.[^32^, ^40^, ^41^] Since 1,4,5,8‐Tetrakis(phenylamino)anthracene‐9,10‐dione (TPAD) is known as a NIR dye, Yan et al. utilized amine‐functionalized TPAD as building units to synthesize a TPAD‐COF (Figure 5a).^[40]^ TPAD‐COF showed a broad light absorption covering the entire UV/Vis and NIR regions as well as superhydrophilicity. Combined with PTFE substrates, the TPAD‐COF‐based evaporators showed a high energy conversion efficiency of 94 % and SVG rate of 1.42 kg m^−2^ h^−1^ under one sun. Ding et al. synthesized a squaraine (SQ)‐linked COF as solar absorber, which also showed broad absorption from 400 to 2400 nm (Figure 5b).^[32]^ Applying melamine foam (MF) as substrates, the SQ‐COF/MF evaporator could generate vapor at 1.35 kg m^−2^ h^−1^ under one sun. Huang et al. incorporated diketopyrrolopyrrole (DPP) into a COF backbone, which again yielded broad light absorption (Figure 5c).^[41]^ The evaporators generated from DPP‐COF with a PVA gel showed 93.2 % efficiency and SVG rate of 2.5 kg m^−2^ h^−1^ under one sun.

Even though, COFs can be applied as photothermal materials, they so far do not perform as well as carbon materials like graphene and CNTs for SVG applications. However, the tunable porous and chemical structure of COFs make them also interesting for other functional parts in solar vapor generators. Our group recently developed free‐standing COF/graphene dual‐region hydrogels (CGHs) as solar evaporators by a one‐step hydrothermal process.^[18b]^ Applying 1,3,5‐triformylphloroglucinol and 2,5‐diaminobenzenesulfonic acid as monomers, TpPa‐SO_3_H‐COF with hydrophilic sulfonic groups was in situ grown on graphene layers (Figure 5d–f). By changing the COF/graphene concentrations, CGHs could be obtained which contained hydrophobic reduced graphene oxide (rGO) regions and hydrophilic COF‐loaded rGO (COF@rGO) regions in variable ratios. The growth of the COF on the graphene layers enhanced the light‐harvesting ability due to the increased surface roughness (Figure 5g and 5h). Furthermore, by changing the COF amount, the pore size of the hydrogels and the wetting ability was continuously changed, which regulates the saturated water content and the ratio of different water states in the CGHs (Figure 5i). Consequently, by optimizing the COF ratio, the amount of IW can reach a maximum and the corresponding CGH (CGH‐50) displayed a considerable SVG rate of 3.69 kg m^−2^ h^−1^ and ≈92 % light utilizing efficiency under one sun (Figure 5j).

### 3.3 Small Molecule‐Based Evaporators

**Figure 6:**
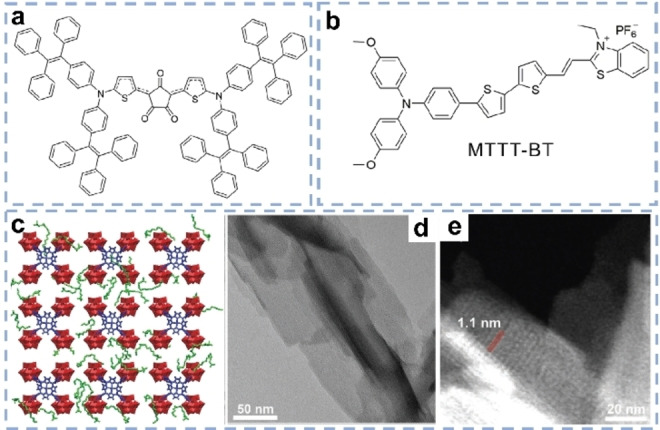
Chemical structure of a) CR‐TPE‐T^[29]^ and b) AIE molecule.^[45]^ c) The structure models of the assembled DNS. d) Transmission electron microscopy (TEM) image of DNS. e) Scanning TEM image of DNS.^[22b]^

Some novel molecules have been lately employed to absorb and convert solar energy in solar evaporators. Croconium molecules, with an intense but narrow absorption band at ≈800 nm, cannot capture sunlight sufficiently. Chen et al. therefore designed a croconium‐based molecule (CR‐TPE‐T) for effective photothermal conversion.^[29]^ The biradical property enhanced the non‐radiative decay for generating heat (Figure 6a). Combined with a PU substrate, the CR‐TPE‐T based evaporators displayed a SVG rate of 1.27 kg m^−2^ h^−1^. Li et al. doped aggregation induced emission (AIE) molecules into 3D fiber aerogels to assemble solar evaporators (Figure 6b).^[45]^ The fiber aerogel supported AIE evaporator could be rapidly heated to 87 °C in 100 s under one sun, which is a remarkable value among so far reported evaporators. AIE‐based evaporators could evaporate water at the rate of 1.43 kg m^−2^ h^−1^ (efficiency 86.5 %). Very recently, phosphomolybdic acid as electron acceptor was combined with porphyrins as electron donors, to assemble 2D dendritic nanosheets (DNS) with enhanced photothermal ability (Figure 6c and d).^[22b]^ The DNS showed well‐ordered channels of ≈1.1 nm, which could enhance water transport via strong capillary force (Figure 6e). Accordingly, the DNS‐based membrane showed a SVG rate of 2.23 kg m^−2^ h^−1^ with energy efficiency of 90.9 % under one sun. Notably, the SVG rate could still reach 1.31 kg m^−2^ h^−1^ even under 0.5 sun.

## 4 Applications

In the solar purification process, a pure water steam is generated through the solar evaporators under sunlight, which can be collected for further usage. SVG has thus be exploited to produce fresh water from seawater, industrial wastewater and domestic sewage. Beyond that, SVG has also been developed for simultaneous power generation.^[12b]^ In the following, some examples for these applications are shown, using the various evaporators described above.

### 4.1 Desalination

**Figure 7:**
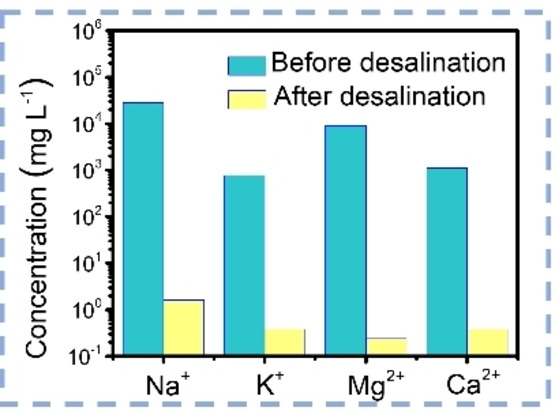
Ion concentrations of simulated seawater before and after desalination applying CGH as evaporator.^[18b]^

Since salt water accounts for 96.5 % of water on earth, desalination is an important way to produce drinking water.^[3]^ As an example, the ion concentrations (Na^+^, K^+^, Ca^2+^, and Mg^2+^) of salt water could be significantly decreased by 4 orders of magnitude after solar desalination tests using the CGH evaporator described above (Figure 7).^[18b]^ It should be however noted, that in practical applications the seawater is transported continuously from bulk water to the evaporation surface, which, due to the high salt concentration of seawater leads to increased salt precipitation, thus hindering further water transportation and decreasing the light absorption.^[24]^ Regularly removing the precipitated salts would however cause high‐cost and less efficient handling in practice. Therefore, systems with anti‐salt clogging abilities, also referred as salt‐free/salt‐rejection abilities, have been developed for sustainable solar desalination. The concept of the anti‐salt clogging ability is to reject the salt crystallization at the evaporating surfaces and in the evaporator channels during brine treatment. For example, natural wood‐based evaporators have been proven to accelerate the ions exchange and thus suppress the salt precipitation by multi‐dimensional water pathways.[^23^, ^24^] Materials with ionic functionalities can also inhibit the salt clogging through selective ion rejection.^[46]^

### 4.2 Wastewater purification

**Figure 8:**
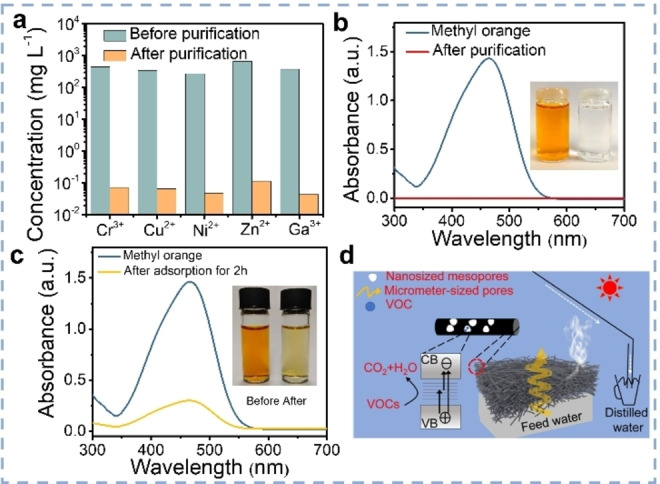
a) Heavy metal ion concentrations before and after solar purification. UV‐vis spectra and photographs (insert) of dye‐contaminated water and purified water applying CGHs through b) solar purification and c) adsorption.^[18a]^ d) VOCs‐intercepting solar distillation enabled by TiO_2−*x*_ NFM.^[47]^

Industrial wastewater often contains heavy metal ions, such as Ga^3+^, Cd^2+^, Cr^3+^, Cu^2+^, Ni^2+^, Pb^2+^, Se^2+^, As^5+^, Zn^2+^, which can be effectively removed via solar evaporation.^[16]^ For example, CGHs showed a removal efficiency of heavy metal ions of ≈99.95 % after solar purification (Figure 8a), which could meet the standard of drinking water from the World Health Organization (WHO).^[18b]^ The removal of typical dye molecules (i.e., methylene orange, methylene blue) has been also broadly studied and could reach almost 100 % efficiency using CGHs for SVG, which is a much better value than when the CGHs are used as adsorbents (Figure 8b and c). As industrial wastewater often also contains VOCs (i.e., benzene, toluene, phenol), their removal during SVG have attracted attention recently.[^30^, ^47^, ^48^] VOCs‐contaminated water cannot be purified during an ordinary solar evaporation, as the VOCs will evaporate as well, additional photocatalysts were employed to degrade the VOCs. Song et al. introduced oxygen‐vacancy‐rich TiO_2−*x*_ into nanofiber membranes (NFM), which provided adequate reactive sites to generate free radicals for VOCs photo‐degradation (Figure 8d).^[47]^ This hybrid membrane can intercept phenol (10 mg L^−1^) with the efficiency of 95 % in the long‐term tests for 30 days. Another intriguing strategy, mentioned above, is to apply MOFs as molecular sieving layers to allow water to evaporate but to intercept VOCs.^[30]^

### 4.3 Sterilization

**Figure 9:**
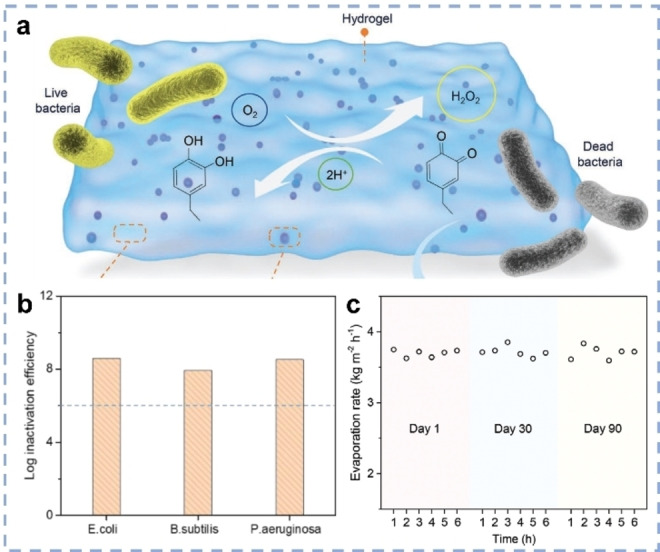
a) Scheme of ABHs for water disinfection. b) Log inactivation of bacteria achieved by ABH‐based SVG. c) Duration test of ABHs after storing in bacteria‐containing river water for 3 months.^[18e]^

Disinfection of drinking water from microorganism pollutants is an important topic. The anti‐bacterial/viral ability of SVG systems was therefore exploited to produce fully purified water.[^2b^, ^14^] Guo et al. developed anti‐bacterial hydrogels (ABHs) modified by catechol and quinone functionalities.^[18e]^ Spontaneous oxidation of catechol groups by O_2_ in both air and water generated O_2_^.−^ and H_2_O_2_, acting as disinfecting agents (Figure 9a). When applying ABHs as solar evaporators to treat water samples from rivers under natural sunlight, the content of bacteria, i.e., *Bacillus subtilis*, *Escherichia coli*, and *Pseudomonas aeruginosa*, in the collected water was reduced to a level below the drinking water standards of WHO (Figure 9b). When ABHs were stored inside the bacteria‐containing river water for 90 days, the SVG rates remained nearly constant, indicating its good stability and durability for long‐term usage (Figure 9c).

### 4.4 Energy Production

**Figure 10:**
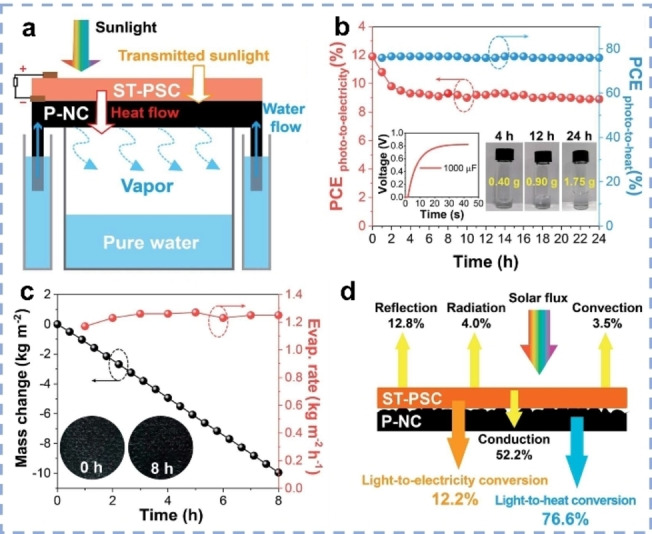
a) Device structure of the PSC/SG hybrid system. b) A long‐term stability test of ST‐PSC/P‐NC over 24 h. c) Solar desalination of ST‐PSC/P‐NC. d) Solar energy budgets of ST‐PSC/P‐NC device.^[12b]^

When thermal energy is localized on the surface of SVG systems for steam generation, energy loss into surroundings and salinity/temperature gradients can be converted to electricity through several energy conversion technologies.^[12b]^ For example, Ji et al. fabricated a solar‐driven water/electricity generator (P‐NC/ST‐PSC), coupling the evaporator (P‐NC) with a semitransparent photovoltaic cell (ST‐PSC). The top solar cell affords the light‐to‐electricity conversion, while the solar evaporator works to convert the transmitted light and conduction heat flow from the solar panels to evaporate the water. The hybrid device gained a superior electrical power output of 122 W m^−2^ and a stable SVG rate of 1.3 kg m^−2^ h^−1^ under one sun (Figure 10a–c).^[12b]^ The total energy efficiency was maximized to 88.8 %, exceeding the pure SVG with 82.6 % efficiency (Figure 10d). Combining power generation and solar evaporation thus opens a new pathway for the utilization of solar energy to meet the increasing demand for clean water and energy.

## 5 Conclusion and Perspectives

**Figure 11:**
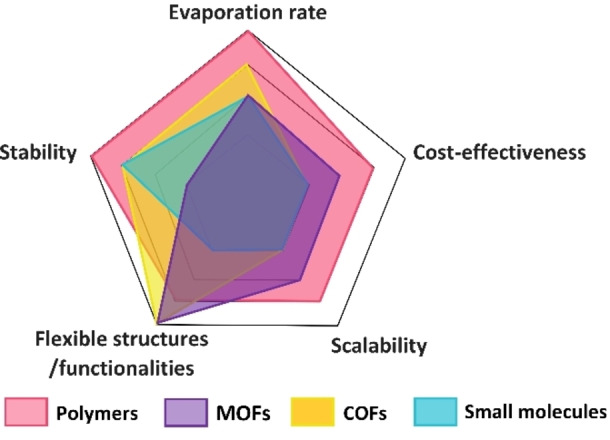
Evaluation of different factors for SVG materials regarding key requirements in practical applications.

The use of solar energy has considerable potential for water purification applications. In this minireview, we describe the recent progress of different materials, such as polymers, MOFs, COFs, and some novel molecules applied as solar water evaporators. Figure 11 provides a qualitative comparison of key factors for their practical application. Polymer materials seem to be currently the most promising materials as they exhibit highest water production yields together with favorable mechanical and chemical stability and scalability, as well as relative low costs. MOFs and COFs lag behind at present, but their very high surface area, tunable pore size and functionality make them still very promising candidates for future SVG systems. Besides, very stable MOFs and COFs have been developed recently and the scalability issue has been solved at least for a range of MOFs. Finally, small molecules for SVGs can be tailored for good photothermal conversion and also the facile processability is an advantage for certain applications. However, they lack porosity and thus have to be composited with other materials and supports. To achieve high SVG performance, the structures of solar evaporators have to be adjusted on several length scales to enhance solar absorption and heat confinement, effective water supply, enlarge the evaporation surface, and lower energy consumption. In 3D porous evaporators, the channel size and the interconnectivity between channels are studied to improve the water supply. The interactions between water and hydrophilic components in solar evaporators are also explored to decrease the energy consumption for vaporization. Some functional systems, such as ionic polymers and wood‐derived substrates, are introduced to prevent salt accumulation in solar distillation. However, the cost, scalability and durability must be taken into consideration for large scale applications. So far, the above‐mentioned evaporation rates are only measured in lab scale, most with an evaporator area of a few square centimeters. Even though, the water yield, especially under weak irradiation, is still too low for large scale practical applications. Also chemical stability, mechanical strength, non‐toxicity as well as low environmental effect of the applied compounds need further attention for future applications. Substantial efforts in this field are expected to develop high‐efficient and practical SVG systems in the future.

## Conflict of interest

The authors declare no conflict of interest.

## Biographical Information

*Sijia Cao received her master degree in 2021 from Humboldt Universität zu Berlin. In Master's period, she studied COF/graphene hybrid materials for environmental applications at the Technische Universität Berlin with Prof. Arne Thomas. Afterwards, she started her Ph.D. research in the group of Prof. Yan Lu in Helmholtz‐Zentrum Berlin für Materialien und Energie, Germany. Her research focuses on COF‐based functional materials for energy applications*.

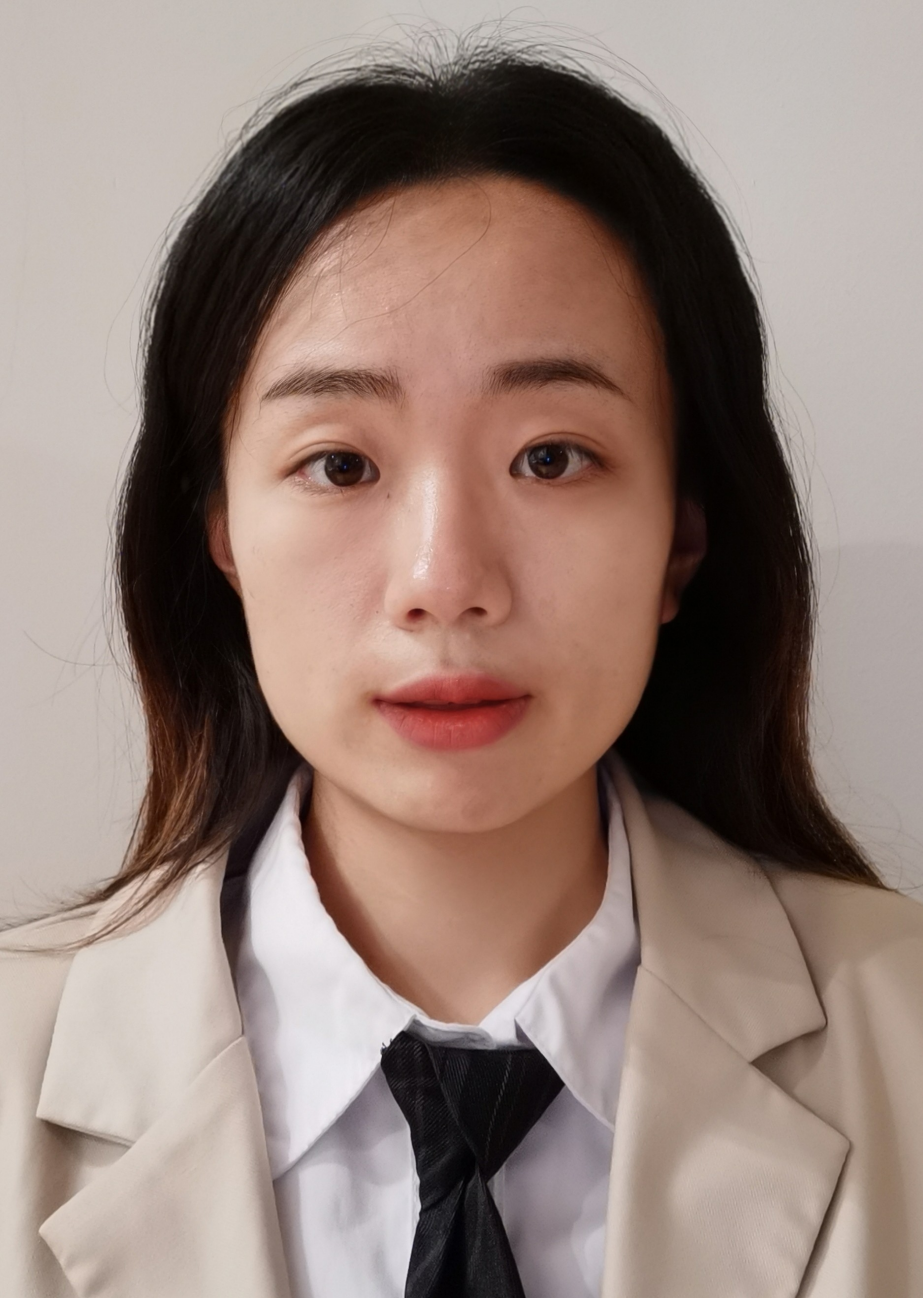

## Biographical Information

*Changxia Li received her Ph.D. in inorganic chemistry from Beijing Institute of Technology, China under the supervision of Prof. Liangti Qu in 2018. From 2019 to 2020, she carried out postdoctoral research with Prof. Arne Thomas at the Technische Universität Berlin, Germany. Then, she joined the group of Prof. Freddy Kleitz at the University of Vienna, Austria as a postdoctoral researcher. Her research interests focus on the design and synthesis of COF/graphene‐based functional materials for energy and environment applications*.

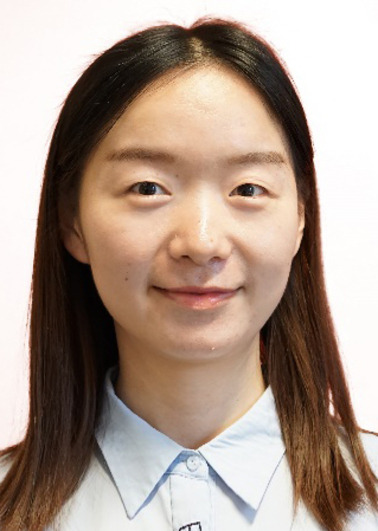

## Biographical Information

*Arne Thomas received his Ph.D. from the Max Planck Institute of Colloids and Interfaces in Potsdam, Germany. After a postdoctoral stay at the University of California, Santa Barbara, as an AvH fellow, he rejoined the MPIKGF as a group leader. In 2009, he became a professor at the Technische Universität Berlin, where he is leading the Department of Functional Materials. His research focuses on porous materials, from mesoporous inorganic to microporous organic materials*.

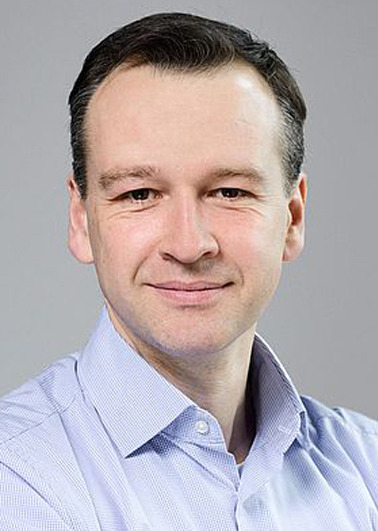

## References

[1a] T. Pointet , LHB Hydrosci. J. 2022, 108, 2090867;

[1b] A. Boretti , L. Rosa , Npj Clean Water 2019, 2, 15;

[1c] M. M. Mekonnen , A. Y. Hoekstra , Sci. Adv. 2016, 2, e1500323; 26933676 10.1126/sciadv.1500323 PMC4758739

[1d] T. Rume , S. M. D. Islam , Heliyon 2020, 6, e04965; 32964165 10.1016/j.heliyon.2020.e04965 PMC7498239

[1e] X. Li , W. A. Mitch , Environ. Sci. Technol. 2018, 52, 1681–1689. 29283253 10.1021/acs.est.7b05440

[2a] S. Liyanaarachchi , L. Shu , S. Muthukumaran , V. Jegatheesan , K. Baskaran , Rev. Environ. Sci. Bio/Technol. 2014, 13, 203–214;

[2b] S. Santoro , A. H. Avci , A. Politano , E. Curcio , Chem. Soc. Rev. 2022, 51, 6087–6152. 35789347 10.1039/d0cs00097c

[3] A. Omarova , K. Tussupova , P. Hjorth , M. Kalishev , R. Dosmagambetova , Int. J. Environ. Res. Public Health 2019, 16, 688. 30813591 10.3390/ijerph16050688 PMC6427320

[4a] S. Shalaby , A. Kabeel , H. Abosheiasha , M. Elfakharany , E. El-Bialy , A. Shama , R. Vidic , J. Cleaner Prod. 2022, 366, 132949;

[4b] M. Gao , C. K. Peh , F. L. Meng , G. W. Ho , Small Methods 2021, 5, 2001200. 10.1002/smtd.202001200 34928082

[5a] D. Lapotko , Opt. Express 2009, 17, 2538; 19219157 10.1364/oe.17.002538

[5b] O. Neumann , A. S. Urban , J. Day , S. Lal , P. Nordlander , N. J. Halas , ACS Nano 2013, 7, 42–49. 23157159 10.1021/nn304948h

[6a] X. Hu , J. Zhu , Adv. Funct. Mater. 2020, 30, 1907234;

[6b] H. Liu , Z. Huang , K. Liu , X. Hu , J. Zhou , Adv. Energy Mater. 2019, 9, 1900310;

[6c] Y. Xia , Y. Kang , Z. Wang , S. Yuan , Y. Li , L. Gao , H. Wang , X. Zhang , J. Mater. Chem. A 2021, 9, 6612–6633;

[6d] P. Tao , G. Ni , C. Song , W. Shang , J. Wu , J. Zhu , G. Chen , T. Deng , Nat. Energy 2018, 3, 1031–1041;

[6e] X. Min , B. Zhu , B. Li , J. Li , J. Zhu , Acc. Mater. Res. 2021, 2, 198–209.

[7a] Z. Xie , Y. Duo , Z. Lin , T. Fan , C. Xing , L. Yu , R. Wang , M. Qiu , Y. Zhang , Y. Zhao , X. Yan , H. Zhang , Adv. Sci. 2020, 7, 1902236; 10.1002/advs.201902236 PMC7055570 32154070

[7b] L. Zhu , M. Gao , C. K. N. Peh , G. W. Ho , Mater. Horiz. 2018, 5, 323–343;

[7c] S. Y. Tee , E. Ye , C. P. Teng , Y. Tanaka , K. Y. Tang , K. Y. Win , M. Han , Nanoscale 2021, 13, 14268–14286; 34473186 10.1039/d1nr04197e

[7d] Y. Lin , H. Xu , X. Shan , Y. Di , A. Zhao , Y. Hu , Z. Gan , J. Mater. Chem. A 2019, 7, 19203–19227;

[7e] M. Gao , C. K. Peh , F. L. Meng , G. W. Ho , Small Methods 2021, 5, 2001200; 10.1002/smtd.202001200 34928082

[7f] C. Shao , Y. Zhao , L. Qu , ChemNanoMat 2020, 6, 1028–1048.

[8a] F. Zhao , Y. Guo , X. Zhou , W. Shi , G. Yu , Nat. Rev. Mater. 2020, 5, 388–401;

[8b] Y. Guo , G. Yu , Acc. Mater. Res. 2021, 2, 374–384.

[9a] Z. Xu , Z. Li , Y. Jiang , G. Xu , M. Zhu , W. Law , K. Yong , Y. Wang , C. Yang , B. Dong , F. Xing , J. Mater. Chem. A 2020, 8, 25571–25600;

[9b] Z. Li , X. Xu , X. Sheng , P. Lin , J. Tang , L. Pan , Y. V. Kaneti , T. Yang , Y. Yamauchi , ACS Nano 2021, 15, 12535–12566. 34279074 10.1021/acsnano.1c01590

[10] Y. Guo , X. Zhou , F. Zhao , J. Bae , B. Rosenberger , G. Yu , ACS Nano 2019, 13, 7913–7919. 31251027 10.1021/acsnano.9b02301

[11] L. Wu , Z. Dong , Z. Cai , T. Ganapathy , N. Fang , C. Li , C. Yu , Y. Zhang , Y. Song , Nat. Commun. 2020, 11, 521. 31988314 10.1038/s41467-020-14366-1 PMC6985111

[12a] F. Zhao , X. Zhou , Y. Shi , X. Qian , M. Alexander , X. Zhao , S. Mendez , R. Yang , L. Qu , G. Yu , Nat. Nanotechnol. 2018, 13, 489–495; 29610528 10.1038/s41565-018-0097-z

[12b] Q. Ji , N. Li , S. Wang , S. Li , F. Li , L. Yu , P. Murto , X. Xu , J. Mater. Chem. A 2021, 9, 21197–21208;

[12c] Y. Shi , O. Ilic , H. A. Atwater , J. R. Greer , Nat. Commun. 2021, 12, 2797; 33990601 10.1038/s41467-021-23174-0 PMC8121874

[12d] L. Zhang , B. Tang , J. Wu , R. Li , P. Wang , Adv. Mater. 2015, 27, 4889–4894; 26184454 10.1002/adma.201502362

[12e] Z. Xie , J. Zhu , L. Zhang , ACS Appl. Mater. Interfaces 2021, 13, 9027–9035. 33577283 10.1021/acsami.0c22917

[13a] Y. Guo , F. Zhao , X. Zhou , Z. Chen , G. Yu , Nano Lett. 2019, 19, 2530–2536; 30836007 10.1021/acs.nanolett.9b00252

[13b] Y. Guo , X. Zhao , F. Zhao , Z. Jiao , X. Zhou , G. Yu , Energy Environ. Sci. 2020, 13, 2087–2095.

[14] Z. Tang , D. Ma , Q. Chen , Y. Wang , M. Sun , Q. Lian , J. Shang , P. K. Wong , C. He , D. Xia , T. Wang , J. Hazard. Mater. 2022, 437, 129373. 35728326 10.1016/j.jhazmat.2022.129373

[15a] Q. Jiang , L. Tian , K. Liu , S. Tadepalli , R. Raliya , P. Biswas , R. R. Naik , S. Singamaneni , Adv. Mater. 2016, 28, 9400–9407; 27432591 10.1002/adma.201601819

[15b] T. Gao , Y. Li , C. Chen , Z. Yang , Y. Kuang , C. Jia , J. Song , E. M. Hitz , B. Liu , H. Huang , J. Yu , B. Yang , L. Hu , Small Methods 2019, 3, 1800176;

[15c] H. Huang , L. Zhao , Q. Yu , P. Lin , J. Xu , X. Yin , S. Chen , H. Wang , L. Wang , ACS Appl. Mater. Interfaces 2020, 12, 11204–11213. 32030971 10.1021/acsami.9b22338

[16] Y. Guo , H. Lu , F. Zhao , X. Zhou , W. Shi , G. Yu , Adv. Mater. 2020, 32, 1907061. 10.1002/adma.201907061 32022974

[17] H. Liang , Q. Liao , N. Chen , Y. Liang , G. Lv , P. Zhang , B. Lu , L. Qu , Angew. Chem. Int. Ed. 2019, 58, 19041–19046; 10.1002/anie.201911457 31605566

[18a] C. Li , J. Yang , P. Pachfule , S. Li , M. Y. Ye , J. Schmidt , A. Thomas , Nat. Commun. 2020, 11, 4712; 32948768 10.1038/s41467-020-18427-3 PMC7501297

[18b] C. Li , S. Cao , J. Lutzki , J. Yang , T. Konegger , F. Kleitz , A. Thomas , J. Am. Chem. Soc. 2022, 144, 3083–3090; 35138088 10.1021/jacs.1c11689

[18c] X. Zhou , F. Zhao , Y. Guo , B. Rosenberger , G. Yu , Sci. Adv. 2019, 5, 5484; 10.1126/sciadv.aaw5484 PMC6599166 31259243

[18d] X. Zhou , Y. Guo , F. Zhao , W. Shi , G. Yu , Adv. Mater. 2020, 32, 2007012; 10.1002/adma.202007012 33184918

[18e] Y. Guo , C. M. Dundas , X. Zhou , K. P. Johnston , G. Yu , Adv. Mater. 2021, 33, 2102994. 10.1002/adma.202102994 34292641

[19a] K. Kudo , J. Ishida , G. Syuu , Y. Sekine , T. Ikeda-Fukazawa , J. Chem. Phys. 2014, 140, 044909; 25669585 10.1063/1.4862996

[19b] Y. Sekine , T. Ikeda-Fukazawa , J. Chem. Phys. 2009, 130, 034501. 19173525 10.1063/1.3058616

[20a] X. Xu , J. Chen , J. Zhou , B. Li , Adv. Mater. 2018, 30, 1705544; 10.1002/adma.201705544 29573283

[20b] L. Tan , B. Tan , Chem. Soc. Rev. 2017, 46, 3322–3356. 28224148 10.1039/c6cs00851h

[21a] Y. Guo , L. S. Vasconcelos , N. Manohar , J. Geng , K. P. Johnston , G. Yu , Angew. Chem. Int. Ed. 2022, 61, e202114074; 10.1002/anie.202114074 34780100

[21b] C. Li , L. Fan , R. Zhu , X. Li , P. Wen , X. Zhao , G. Wang , J. Zou , F. Kim , ACS Appl. Energ. Mater. 2020, 3, 9216–9225.

[22a] W. Tu , Z. Wang , Q. Wu , H. Huang , Y. Liu , M. Shao , B. Yao , Z. Kang , J. Mater. Chem. A 2020, 8, 10260–10268;

[22b] Y. Zhou , Q. Lu , Q. Liu , H. Yang , J. Liu , J. Zhuang , W. Shi , X. Wang , Adv. Funct. Mater. 2022, 32, 2112159.

[23] S. He , C. Chen , Y. Kuang , R. Mi , Y. Liu , Y. Pei , W. Kong , W. Gan , H. Xie , E. Hitz , C. Jia , X. Chen , A. Gong , J. Liao , J. Li , Z. J. Ren , B. Yang , S. Das , L. Hu , Energy Environ. Sci. 2019, 12, 1558–1567.

[24] Y. Kuang , C. Chen , S. He , E. M. Hitz , Y. Wang , W. Gan , R. Mi , L. Hu , Adv. Mater. 2019, 31, 1900498. 10.1002/adma.201900498 30989752

[25a] A. W. Carpenter , C. F. de Lannoy , M. R. Wiesner , Environ. Sci. Technol. 2015, 49, 5277–5287; 25837659 10.1021/es506351r PMC4544834

[25b] S. Cao , P. Rathi , X. Wu , D. Ghim , Y. Jun , S. Singamaneni , Adv. Mater. 2021, 33, 2000922. 10.1002/adma.202000922 32537817

[26] W. Li , Z. Li , K. Bertelsmann , D. Fan , Adv. Mater. 2019, 31, 1900720. 10.1002/adma.201900720 31134676

[27] S. Li , Y. He , Y. Guan , X. Liu , H. Liu , M. Xie , L. Zhou , C. Wei , C. Yu , Y. Chen , ACS Appl. Polym. Mater. 2020, 2, 4581–4591.

[28] L. Chen , D. Li , Y. Wang , C. Duan , Nanoscale 2019, 11, 11121–11127. 31070200 10.1039/c8nr09080g

[29] G. Chen , J. Sun , Q. Peng , Q. Sun , G. Wang , Y. Cai , X. Gu , Z. Shuai , B. Tang , Adv. Mater. 2020, 32, 1908537. 10.1002/adma.201908537 32519356

[30] Y. Peng , X. Wei , Y. Wang , W. Li , S. Zhang , J. Jin , ACS Nano 2022, 16, 8329–8337. 35549179 10.1021/acsnano.2c02520

[31] W. Cui , C. Zhang , R. Liang , J. Liu , J. Qiu , ACS Appl. Mater. Interfaces 2021, 13, 31561–31568. 34192870 10.1021/acsami.1c04419

[32] N. Ding , T. Zhou , W. Weng , Z. Lin , S. Liu , P. Maitarad , C. Wang , J. Guo , Small 2022, 18, 2201275. 10.1002/smll.202201275 35585681

[33] Z. Li , X. Ma , D. Chen , X. Wan , X. Wang , Z. Fang , X. Peng , Adv. Sci. 2021, 8, 2004552. 10.1002/advs.202004552 PMC8025007 33854905

[34] X. Xu , S. Ozden , N. Bizmark , C. B. Arnold , S. S. Datta , R. D. Priestley , Adv. Mater. 2021, 33, 2007833. 10.1002/adma.202007833 33786873

[35a] Z. Xia , Y. Zhao , S. B. Darling , Adv. Mater. Interfaces 2020, 8, 2001507;

[35b] C. Li , W. Ju , S. Vijay , J. Timoshenko , K. Mou , D. A. Cullen , J. Yang , X. Wang , P. Pachfule , S. Bruckner , H. S. Jeon , F. T. Haase , S. C. Tsang , C. Rettenmaier , K. Chan , B. R. Cuenya , A. Thomas , P. Strasser , Angew. Chem. Int. Ed. 2022, 61, e202114707; 10.1002/anie.202114707 PMC9306911 35102658

[35c] C. Li , P. Guggenberger , S. Han , W. Ding , F. Kleitz , Angew. Chem. Int. Ed. 2022, 61, e202206564; 10.1002/anie.202206564 PMC9541632 35639272

[35d] V. Bon , I. Senkovska , J. D. Evans , M. Wöllner , M. Hölzel , S. Kaskel , J. Mater. Chem. A 2019, 7, 12681–12690.

[36] H. Kim , S. Yang , S. Rao , S. Narayanan , E. A. Kapustin , H. Furukawa , A. S. Umans , O. M. Yaghi , E. N. Wang , Science 2017, 356, 430–434. 28408720 10.1126/science.aam8743

[37] H. Furukawa , K. E. Cordova , M. O′Keeffe , O. M. Yaghi , Science 2013, 341, 1230444. 23990564 10.1126/science.1230444

[38] X. Ma , Z. Deng , Z. Li , D. Chen , X. Wan , X. Wang , X. Peng , J. Mater. Chem. A 2020, 8, 22728–22735.

[39] Q. Qian , P. A. Asinger , M. J. Lee , G. Han , K. Mizrahi Rodriguez , S. Lin , F. M. Benedetti , A. X. Wu , W. Chi , Z. P. Smith , Chem. Rev. 2020, 120, 8161–8266. 32608973 10.1021/acs.chemrev.0c00119

[40] X. Yan , S. Lyu , X. Xu , W. Chen , P. Shang , Z. Yang , G. Zhang , W. Chen , Y. Wang , L. Chen , Angew. Chem. Int. Ed. 2022, 61, e202201900; 10.1002/anie.202201900 35235246

[41] Z. Huang , Y. Luo , W. Geng , Y. Wan , S. Li , C. Lee , Small Methods 2021, 5, 2100036. 10.1002/smtd.202100036 34928098

[42a] M. S. Lohse , T. Bein , Adv. Funct. Mater. 2018, 28, 1705553;

[42b] R. Liu , K. Tan , Y. Gong , Y. Chen , Z. Li , S. Xie , T. He , Z. Lu , H. Yang , D. Jiang , Chem. Soc. Rev. 2021, 50, 120–242. 33283811 10.1039/d0cs00620c

[43] W. Cui , C. Zhang , R. Liang , J. Qiu , J. Mater. Chem. A 2021, 9, 25611–25620.

[44] Z. Xia , H. Yang , Z. Chen , R. Z. Waldman , Y. Zhao , C. Zhang , S. N. Patel , S. B. Darling , Adv. Mater. Interfaces 2019, 6, 1900254.

[45] H. Li , H. Wen , J. Li , J. Huang , D. Wang , B. Tang , ACS Appl. Mater. Interfaces 2020, 12, 26033–26040. 32407616 10.1021/acsami.0c06181

[46a] F. Wang , Z. Hu , Y. Fan , W. Bai , S. Wu , H. Sun , Z. Zhu , W. Liang , A. Li , Macromol. Rapid Commun. 2021, 42, 2000536; 10.1002/marc.202000536 33241568

[46b] F. Wang , Y. Su , Y. Li , D. Wei , H. Sun , Z. Zhu , W. Liang , A. Li , ACS Appl. Energ. Mater. 2020, 3, 8746–8754.

[47] C. Song , D. Qi , Y. Han , Y. Xu , H. Xu , S. You , W. Wang , C. Wang , Y. Wei , J. Ma , Environ. Sci. Technol. 2020, 54, 9025–9033. 32589018 10.1021/acs.est.9b07903

[48] J. Deng , S. Xiao , B. Wang , Q. Li , G. Li , D. Zhang , H. Li , ACS Appl. Mater. Interfaces 2020, 12, 51537–51545. 33161716 10.1021/acsami.0c15694

